# *catena*-Poly[[bis­(methanol-κ*O*)bis­(pyridine-κ*N*)nickel(II)]-μ-tetra­fluoro­terephthalato-κ^2^ *O*:*O*′]

**DOI:** 10.1107/S1600536808016619

**Published:** 2008-06-07

**Authors:** Chang-Ge Zheng, Jian-Quan Hong, Jie Zhang, Chao Wang

**Affiliations:** a School of Chemical and Materials Engineering, Jiangnan University, 1800 Lihu Road, Wuxi, Jiangsu Province 214122, People’s Republic of China

## Abstract

In the title compound, [Ni(C_8_F_4_O_4_)(C_5_H_5_N)_2_(CH_4_O)_2_]_*n*_, the Ni^II^ ion is located on an inversion center and is coordinated by four O atoms [Ni—O = 2.079 (4) Å] from two tetra­fluoro­terephthalate ligands and two methanol mol­ecules, and by two N atoms [Ni—N = 2.127 (4) Å] from two pyridine ligands in a distorted octa­hedral geometry. The Ni^II^ ions are connected *via* the tetra­fluoro­terephthalate anions into a one-dimensional chain running along the crystallographic [011] direction.

## Related literature

For useful applications of supra­molecular coordination polymers, see: Janiak (2003 ▶); Rao *et al.* (2004 ▶); James (2003 ▶); Dietzel *et al.* (2005 ▶); Zhang *et al.* (2007 ▶). For related crystal structures, see: Kim *et al.* (2003 ▶); Go *et al.* (2004 ▶); Wang *et al.* (2003 ▶); Śledź *et al.* (2001 ▶); Li *et al.* (2003 ▶); Rosi *et al.* (2005 ▶).
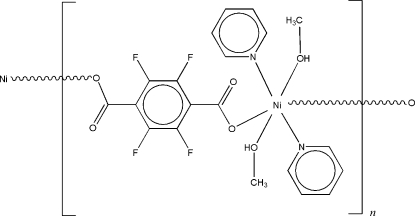


## Experimental

### 

#### Crystal data

[Ni(C_8_F_4_O_4_)(C_5_H_5_N)_2_(CH_4_O)_2_]
*M* *_r_* = 517.07
Triclinic, 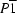

*a* = 7.9159 (7) Å
*b* = 8.8846 (8) Å
*c* = 9.0219 (14) Å
α = 100.442 (9)°
β = 101.559 (9)°
γ = 114.396 (6)°
*V* = 540.78 (11) Å^3^
*Z* = 1
Mo *K*α radiation
μ = 0.97 mm^−1^
*T* = 273 (2) K
0.15 × 0.12 × 0.10 mm

#### Data collection

Bruker APEXII CCD area-detector diffractometer
Absorption correction: multi-scan (*SADABS*; Sheldrick, 2000 ▶) *T*_min_ = 0.868, *T*_max_ = 0.909
3004 measured reflections
1901 independent reflections
1210 reflections with *I* > 2σ(*I*)
*R*_int_ = 0.045

#### Refinement

*R*[*F* ^2^ > 2σ(*F* ^2^)] = 0.065
*wR*(*F* ^2^) = 0.168
*S* = 1.03
1901 reflections
151 parameters
2 restraints
H-atom parameters constrained
Δρ_max_ = 0.67 e Å^−3^
Δρ_min_ = −0.47 e Å^−3^

### 

Data collection: *APEX2* (Bruker, 2005 ▶); cell refinement: *APEX2*; data reduction: *APEX2*; program(s) used to solve structure: *SIR97* (Altomare *et al.*, 1999 ▶); program(s) used to refine structure: *SHELXL97* (Sheldrick, 2008 ▶); molecular graphics: *PLATON* (Spek, 2003 ▶); software used to prepare material for publication: *SHELXL97*.

## Supplementary Material

Crystal structure: contains datablocks I. DOI: 10.1107/S1600536808016619/cv2417sup1.cif

Structure factors: contains datablocks I. DOI: 10.1107/S1600536808016619/cv2417Isup2.hkl

Additional supplementary materials:  crystallographic information; 3D view; checkCIF report

## Figures and Tables

**Table 1:** Hydrogen-bond geometry (Å, °)

*D*—H⋯*A*	*D*—H	H⋯*A*	*D*⋯*A*	*D*—H⋯*A*
O3—H3⋯O2	0.92	1.74	2.581 (6)	151

**Fig. 1.**
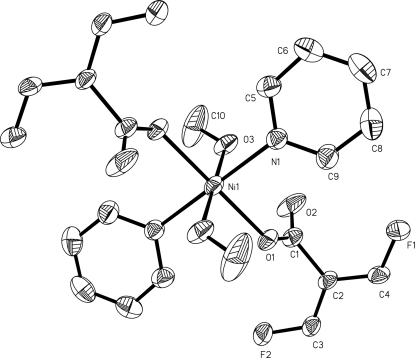
A portion of the crystal structure of (I) showing the atomic numbering and 30% probability displacement ellipsoids. The unlabelled atoms are related with the labelled ones by symmetry operation (-x, -y, -z). H atoms omitted for clarity.

**Fig. 2.**
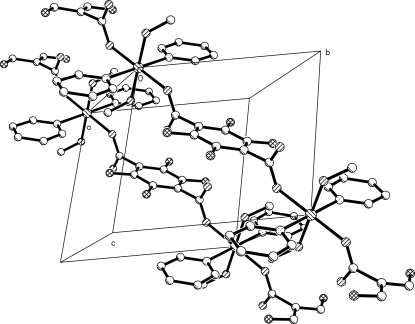
Supplementary figure.

## supplementary crystallographic information

### Comment

Supramolecular coordination polymers have attracted considerable interest recently due to their intriguing network topologies and their potential applications as gas storage systems, sensors, catalysis, ion exchange materials and magnetic materials (Janiak, 2003; Rao *et al.*, 2004; James, 2003; Dietzel *et al.*, 2005). Chemical modification with the organic ligand can be carried out for constructing materials of different properties. Some research work in computational study suggests that adsorption property in gas storage can be improved with electronegative atoms in the organic linkers or frameworks (Zhang *et al.*, 2007). New topologies with favorable properties will be achieved by introducing some strong electronegative atoms (*e.g.* Halogen atoms) to in the aromatic ring.

The one-dimensional linear electronically neutral chains of the title nickel(II) complex, (I) (Fig. 1), crystallizes in the triclinic space group P1. The tetrafluoroterephthalate ligands are coordinated to nickel(II) ion in one monodentate pattern. In the octahedron unit, two O atoms from the tetrafluoroterephthalate ligands and two N atoms from pyridine molecules form the equatorial plane. The axial positions are occupied by O atoms from two methanol molecules with a O—Ni—O angle of 180.0 (3)°. The equatorial plane and pyridyl ring form a dihedral angle of 15.2 (1)°. The Ni—O bond lengths are 2.079 (3) and 2.079 (4) Å and agree well with the reported values in related structures (Kim *et al.*, 2003; Go *et al.*, 2004). Both of the Ni—N bond lengths are 2.127 (4) Å, which are comparable with reported values in the similar complexes (Wang *et al.*, 2003; Li *et al.*, 2003). In the aromatic ring system, the bond lengths and bond angles are slightly larger than that in reported terephthalaic acid (Śledź *et al.*, 2001). In addition, the hydroxyl groups of methanol molecules act as donors in O—H···O hydrogen bonds (Table 1).

### Experimental

All the reagents and solvents employed were commercially available, and tetrafluoroterephthalaic acid was purified by recrystallization. The title compound was prepared according to the literature procedure of Rosi *et al.* (2005).

Tetrafluoroterephthalaic acid (0.0714 g, 0.30 mmol) and Ni(NO_3_)_2_.6H_2_O (0.0436 g, 0.15 mmol) were placed in a small vial and dissolved in a mixture of methanol (3 ml) and acetonitrile (3 ml) at room temperature. The vial was then placed in a larger vial containing pyridine (4 ml), which was sealed and left undisturbed for 7 d at room temperature. The resulting green block-shaped crystals were collected by filtration, washed with methanol (3 ml), and air dried to give the title complex (0.06 g, 77% yield). Elemental analysis (%) calcd. for C_20_H_18_F_4_N_2_NiO_6_: C, 46.45%; H, 3.51%; N, 5.42%; Found: C,46.48%; H, 3.55%; N, 5.38%.

### Refinement

All the other H atoms were placed in geometrically idealized positions and constrained to ride on their parent atoms with C—H and O—H distances of 0.91–0.97 Å, and *U*_iso_(H) = 1.2–1.5 times of those of their parent atoms (Å^2^).

### Crystal data

**Table:**

[Ni(C_8_F_4_O_4_)(C_5_H_5_N)_2_(CH_4_O)_2_]	*Z* = 1
*M**_r_* = 517.07	*F*_000_ = 264
Triclinic, *P*1	*D*_x_ = 1.588 Mg m^−^^3^
Hall symbol: -P1	Mo *K*α radiation λ = 0.71073 Å
*a* = 7.9159 (7) Å	Cell parameters from 409 reflections
*b* = 8.8846 (8) Å	θ = 3.7–18.2º
*c* = 9.0219 (14) Å	µ = 0.97 mm^−^^1^
α = 100.442 (9)º	*T* = 273 (2) K
β = 101.559 (9)º	Block, green
γ = 114.396 (6)º	0.15 × 0.12 × 0.10 mm
*V* = 540.78 (11) Å^3^	

### Data collection

**Table:**

Bruker APEXII CCD area-detector diffractometer	1901 independent reflections
Radiation source: fine-focus sealed tube	1210 reflections with *I* > 2σ(*I*)
Monochromator: graphite	*R*_int_ = 0.045
*T* = 273(2) K	θ_max_ = 25.0º
φ and ω scans	θ_min_ = 3.7º
Absorption correction: multi-scan(SADABS; Sheldrick, 2000)	*h* = −9→9
*T*_min_ = 0.868, *T*_max_ = 0.909	*k* = −8→10
3004 measured reflections	*l* = −10→10

### Refinement

**Table:**

Refinement on *F*^2^	2 restraints
Least-squares matrix: full	H-atom parameters constrained
*R*[*F*^2^ > 2σ(*F*^2^)] = 0.065	*w* = 1/[σ^2^(*F*_o_^2^) + (0.076*P*)^2^ + 0.2195*P*], *P* = (*F*_o_^2^ + 2*F*_c_^2^)/3
*wR*(*F*^2^) = 0.168	(Δ/σ)_max_ < 0.001
*S* = 1.04	Δρ_max_ = 0.67 e Å^−^^3^
1901 reflections	Δρ_min_ = −0.46 e Å^−^^3^
151 parameters	Extinction correction: none

### Special details

**Table:**

Geometry. All e.s.d.'s (except the e.s.d. in the dihedral angle between two l.s. planes) are estimated using the full covariance matrix. The cell e.s.d.'s are taken into account individually in the estimation of e.s.d.'s in distances, angles and torsion angles; correlations between e.s.d.'s in cell parameters are only used when they are defined by crystal symmetry. An approximate (isotropic) treatment of cell e.s.d.'s is used for estimating e.s.d.'s involving l.s. planes.
Refinement. Refinement of *F*^2^ against ALL reflections. The weighted *R*–factor *wR* and goodness of fit *S* are based on *F*^2^, conventional *R*–factors *R* are based on *F*, with *F* set to zero for negative *F*^2^. The threshold expression of *F*^2^ > σ(*F*^2^) is used only for calculating *R*-factors(gt) *etc*. and is not relevant to the choice of reflections for refinement. *R*–factors based on *F*^2^ are statistically about twice as large as those based on *F*, and *R*– factors based on ALL data will be even larger.

### Fractional atomic coordinates and isotropic or equivalent isotropic displacement parameters (Å^2^)

**Table:**

	*x*	*y*	*z*	*U*_iso_*/*U*_eq_	
Ni1	0.0000	0.0000	0.0000	0.0463 (4)	
O1	−0.0204 (5)	0.1748 (4)	0.1738 (4)	0.0514 (10)	
O2	0.2732 (7)	0.2924 (6)	0.3536 (5)	0.0893 (16)	
O3	0.2791 (6)	0.0643 (5)	0.1351 (4)	0.0642 (12)	
H3	0.2848	0.1228	0.2320	0.096*	
N1	0.1405 (7)	0.2043 (5)	−0.0943 (5)	0.0495 (12)	
F1	0.2568 (6)	0.6330 (4)	0.3438 (4)	0.0798 (12)	
F2	−0.1649 (6)	0.1587 (4)	0.4752 (4)	0.0770 (11)	
C1	0.1084 (10)	0.2749 (7)	0.3031 (7)	0.0525 (14)	
C2	0.0498 (8)	0.3903 (7)	0.4030 (6)	0.0476 (13)	
C3	−0.0823 (9)	0.3286 (7)	0.4841 (6)	0.0523 (14)	
C4	0.1304 (8)	0.5655 (7)	0.4223 (6)	0.0538 (15)	
C5	0.2384 (9)	0.1849 (8)	−0.1928 (7)	0.0636 (16)	
H5	0.2395	0.0793	−0.2204	0.076*	
C6	0.3374 (10)	0.3084 (10)	−0.2565 (8)	0.0781 (19)	
H6	0.4006	0.2858	−0.3274	0.094*	
C7	0.3426 (10)	0.4657 (10)	−0.2148 (8)	0.078 (2)	
H7	0.4120	0.5537	−0.2543	0.093*	
C8	0.2427 (11)	0.4911 (9)	−0.1129 (8)	0.079 (2)	
H8	0.2415	0.5964	−0.0830	0.095*	
C9	0.1441 (10)	0.3574 (8)	−0.0557 (7)	0.0643 (16)	
H9	0.0767	0.3754	0.0133	0.077*	
C10	0.3668 (15)	−0.0333 (11)	0.1774 (13)	0.148 (5)	
H10A	0.3276	−0.0734	0.2628	0.222*	
H10B	0.5059	0.0361	0.2107	0.222*	
H10C	0.3283	−0.1308	0.0882	0.222*	

### Atomic displacement parameters (Å^2^)

**Table:**

	*U*^11^	*U*^22^	*U*^33^	*U*^12^	*U*^13^	*U*^23^
Ni1	0.0591 (8)	0.0449 (6)	0.0396 (6)	0.0293 (5)	0.0189 (5)	0.0065 (4)
O1	0.066 (3)	0.050 (2)	0.040 (2)	0.033 (2)	0.018 (2)	0.0029 (18)
O2	0.078 (3)	0.108 (4)	0.063 (3)	0.056 (3)	0.004 (3)	−0.029 (3)
O3	0.069 (3)	0.074 (3)	0.051 (2)	0.044 (2)	0.017 (2)	0.000 (2)
N1	0.056 (3)	0.049 (3)	0.046 (3)	0.026 (2)	0.018 (2)	0.012 (2)
F1	0.103 (3)	0.071 (2)	0.083 (3)	0.040 (2)	0.064 (2)	0.0212 (19)
F2	0.102 (3)	0.051 (2)	0.086 (3)	0.035 (2)	0.052 (2)	0.0126 (18)
C1	0.065 (4)	0.053 (3)	0.041 (3)	0.030 (3)	0.022 (3)	0.005 (3)
C2	0.054 (3)	0.047 (3)	0.038 (3)	0.025 (3)	0.015 (3)	−0.002 (2)
C3	0.064 (4)	0.044 (3)	0.046 (3)	0.027 (3)	0.019 (3)	0.003 (2)
C4	0.062 (4)	0.062 (4)	0.043 (3)	0.031 (3)	0.027 (3)	0.009 (3)
C5	0.076 (4)	0.066 (4)	0.063 (4)	0.039 (3)	0.036 (3)	0.022 (3)
C6	0.072 (4)	0.089 (5)	0.081 (4)	0.032 (4)	0.037 (4)	0.038 (4)
C7	0.067 (4)	0.077 (4)	0.074 (4)	0.014 (4)	0.016 (4)	0.039 (4)
C8	0.097 (5)	0.055 (4)	0.077 (4)	0.031 (4)	0.018 (4)	0.024 (3)
C9	0.086 (4)	0.056 (3)	0.058 (3)	0.036 (3)	0.029 (3)	0.017 (3)
C10	0.141 (8)	0.102 (6)	0.174 (10)	0.092 (7)	−0.036 (7)	−0.014 (6)

### Geometric parameters (Å, °)

**Table:**

Ni1—O3	2.079 (4)	C2—C3	1.381 (7)
Ni1—O3^i^	2.079 (4)	C3—C4^ii^	1.371 (7)
Ni1—O1^i^	2.079 (3)	C4—C3^ii^	1.371 (7)
Ni1—O1	2.079 (3)	C5—C6	1.359 (8)
Ni1—N1^i^	2.127 (4)	C5—H5	0.9300
Ni1—N1	2.127 (4)	C6—C7	1.361 (9)
O1—C1	1.261 (6)	C6—H6	0.9300
O2—C1	1.226 (7)	C7—C8	1.373 (10)
O3—C10	1.375 (8)	C7—H7	0.9300
O3—H3	0.9147	C8—C9	1.379 (9)
N1—C5	1.323 (7)	C8—H8	0.9300
N1—C9	1.329 (7)	C9—H9	0.9300
F1—C4	1.343 (6)	C10—H10A	0.9600
F2—C3	1.354 (6)	C10—H10B	0.9600
C1—C2	1.518 (7)	C10—H10C	0.9600
C2—C4	1.377 (7)		
			
O3—Ni1—O3^i^	180.0 (3)	F2—C3—C4^ii^	118.0 (5)
O3—Ni1—O1^i^	88.71 (14)	F2—C3—C2	119.6 (5)
O3^i^—Ni1—O1^i^	91.29 (14)	C4^ii^—C3—C2	122.3 (5)
O3—Ni1—O1	91.29 (14)	F1—C4—C3^ii^	119.4 (5)
O3^i^—Ni1—O1	88.71 (14)	F1—C4—C2	118.5 (5)
O1^i^—Ni1—O1	180.0 (2)	C3^ii^—C4—C2	122.1 (5)
O3—Ni1—N1^i^	94.29 (17)	N1—C5—C6	124.6 (6)
O3^i^—Ni1—N1^i^	85.71 (17)	N1—C5—H5	117.7
O1^i^—Ni1—N1^i^	89.23 (15)	C6—C5—H5	117.7
O1—Ni1—N1^i^	90.77 (15)	C5—C6—C7	119.0 (7)
O3—Ni1—N1	85.71 (17)	C5—C6—H6	120.5
O3^i^—Ni1—N1	94.29 (17)	C7—C6—H6	120.5
O1^i^—Ni1—N1	90.77 (15)	C6—C7—C8	118.2 (7)
O1—Ni1—N1	89.23 (15)	C6—C7—H7	120.9
N1^i^—Ni1—N1	180.00 (19)	C8—C7—H7	120.9
C1—O1—Ni1	127.3 (4)	C7—C8—C9	118.8 (6)
C10—O3—Ni1	132.9 (5)	C7—C8—H8	120.6
C10—O3—H3	101.1	C9—C8—H8	120.6
Ni1—O3—H3	101.3	N1—C9—C8	123.3 (6)
C5—N1—C9	116.1 (5)	N1—C9—H9	118.3
C5—N1—Ni1	119.8 (4)	C8—C9—H9	118.3
C9—N1—Ni1	124.0 (4)	O3—C10—H10A	109.5
O2—C1—O1	127.9 (5)	O3—C10—H10B	109.5
O2—C1—C2	117.6 (5)	H10A—C10—H10B	109.5
O1—C1—C2	114.4 (5)	O3—C10—H10C	109.5
C4—C2—C3	115.6 (5)	H10A—C10—H10C	109.5
C4—C2—C1	121.7 (5)	H10B—C10—H10C	109.5
C3—C2—C1	122.6 (5)		
			
O3—Ni1—O1—C1	1.8 (5)	Ni1—O1—C1—C2	178.6 (3)
O3^i^—Ni1—O1—C1	−178.2 (5)	O2—C1—C2—C4	69.3 (8)
O1^i^—Ni1—O1—C1	−146 (100)	O1—C1—C2—C4	−108.6 (6)
N1^i^—Ni1—O1—C1	96.1 (5)	O2—C1—C2—C3	−108.6 (7)
N1—Ni1—O1—C1	−83.9 (5)	O1—C1—C2—C3	73.5 (7)
O3^i^—Ni1—O3—C10	142 (100)	C4—C2—C3—F2	−178.3 (5)
O1^i^—Ni1—O3—C10	−48.5 (8)	C1—C2—C3—F2	−0.3 (8)
O1—Ni1—O3—C10	131.5 (8)	C4—C2—C3—C4^ii^	0.1 (9)
N1^i^—Ni1—O3—C10	40.6 (8)	C1—C2—C3—C4^ii^	178.1 (5)
N1—Ni1—O3—C10	−139.4 (8)	C3—C2—C4—F1	−178.6 (5)
O3—Ni1—N1—C5	72.0 (4)	C1—C2—C4—F1	3.4 (8)
O3^i^—Ni1—N1—C5	−108.0 (4)	C3—C2—C4—C3^ii^	−0.1 (9)
O1^i^—Ni1—N1—C5	−16.7 (4)	C1—C2—C4—C3^ii^	−178.2 (5)
O1—Ni1—N1—C5	163.3 (4)	C9—N1—C5—C6	−0.8 (9)
N1^i^—Ni1—N1—C5	−169 (100)	Ni1—N1—C5—C6	−178.2 (5)
O3—Ni1—N1—C9	−105.2 (5)	N1—C5—C6—C7	1.8 (10)
O3^i^—Ni1—N1—C9	74.8 (5)	C5—C6—C7—C8	−1.7 (10)
O1^i^—Ni1—N1—C9	166.2 (5)	C6—C7—C8—C9	0.9 (10)
O1—Ni1—N1—C9	−13.8 (5)	C5—N1—C9—C8	0.0 (9)
N1^i^—Ni1—N1—C9	14 (100)	Ni1—N1—C9—C8	177.2 (5)
Ni1—O1—C1—O2	0.9 (9)	C7—C8—C9—N1	0.0 (10)

Symmetry codes: (i) −*x*, −*y*, −*z*; (ii) −*x*, −*y*+1, −*z*+1.

### Hydrogen-bond geometry (Å, °)

**Table:**

*D*—H···*A*	*D*—H	H···*A*	*D*···*A*	*D*—H···*A*
O3—H3···O2	0.92	1.74	2.581 (6)	151
